# Family members’ reasoning in relation to pleasant environments in nursing homes

**DOI:** 10.1177/14713012221142474

**Published:** 2022-11-25

**Authors:** Marianne Palmgren, Lena Rosenberg, Sophie Nadia Gaber, Karin Johansson

**Affiliations:** Division of Occupational Therapy, Department of Neurobiology, Care Sciences and Society, NVS, 27106 Karolinska Institutet, Stockholm, Sweden; Division of Information Design, School of Innovation, Design and Engineering, 8177 Mälardalen University, Eskilstuna, Sweden; Department of Rehabilitation, School of Health and Welfare, Jönköping University, Jönköping, Sweden; Division of Occupational Therapy, Department of Neurobiology, Care Sciences and Society, NVS, 27106 Karolinska Institutet, Stockholm, Sweden; Division of Occupational Therapy, Department of Neurobiology, Care Sciences and Society, NVS, 27106 Karolinska Institutet, Stockholm, Sweden; Department of Health Care Sciences, Marie Cederschiöld University, Stockholm, Sweden; Division of Occupational Therapy, Department of Neurobiology, Care Sciences and Society, NVS, 27106 Karolinska Institutet, Stockholm, Sweden

**Keywords:** architecture, dementia, design, environment, everyday life, focus groups, place, relatives, residential care facilities, life space

## Abstract

The physical environment plays an important role in how everyday life is shaped and experienced for persons living in nursing homes as well as for the residents’ family and friends. Still, there is a scarcity of research exploring the perspectives of family members of residents regarding everyday life in common areas in nursing homes. In this study, we chose the term, ‘a pleasant place’, with the ambition of remaining open to various ideas and aspects that family members perceive as relevant when reasoning about the nursing home environment. The study aimed to explore how family members of nursing home residents reason in relation to pleasant places in nursing homes. Four focus group sessions were conducted with a total of 14 family members. Data were analysed using qualitative content analysis. The analysis resulted in four themes. ‘A door ajar’, highlighted the importance of a nursing home environment that provides potential opportunities for pleasurable everyday moments. ‘Why does it have to be so ugly?’, revealed how family members perceived institutional logics as guiding the design of the nursing homes, which were misaligned with the logics of a pleasant place. ‘A place to care for?’, emphasised the physical environment as an integrated aspect of care, in terms of being carefully arranged and used with sensitivity. Finally, ‘allegiance to the place’ showed that despite the family members’ recognitions of shortcomings in the nursing home physical environments, their allegiance to the place provided a sense of the nursing home as a pleasant place. The study contributes knowledge regarding the perceived value of the design of the physical environment in nursing homes, particularly in common areas, as an integral aspect of care, and moves beyond the ideas of homelike and non-institutional nursing home environments.

## Background

For older persons living with dementia in nursing homes, the physical environment plays an important role in how everyday life is shaped and experienced (Fleming et al., 2016). The physical environment in nursing homes may also be important to residents’ family and friends (Van Hoof et al., 2016), as a way of framing and shaping the everyday life of someone they care for (Innes et al., 2011), and influencing how they experience their visits and social interactions with residents, staff and other members of the nursing home community (Førsund & Ytrehus, 2018). Earlier research has highlighted the significant role of the physical environment to enhance person-centred care (Edvardsson et al., 2010) which is emphasised in policies and guidelines regarding the care of persons living with dementia (Socialstyrelsen, 2017). According to a person-centred approach to care, the focus is not on the diagnosis but rather on the person, and this includes his or her social network as a valuable resource (Kitwood, 1997). This study acknowledges the social network of nursing home residents, which is typically comprised of family and/or friends.

Nursing home research indicates that both the physical environment (Calkins, 2018; Chaudhury et al., 2018; Fleming et al., 2016; Kenkmann et al., 2017; Torrington, 2009) and ongoing contact with family and friends (Førsund et al., 2016; Graske et al., 2015) are integral to the social wellbeing of residents living with dementia. Existing research on common areas in nursing homes, such as dining rooms, living rooms, hallways, and balconies, has to a large extent focused on physical and cognitive accessibility for residents (Calkins, 2009; Day et al., 2000; Digby & Bloomer, 2014; Fleming et al., 2016; Robinson et al., 2010). A previous study explored how the physical and social environment of nursing homes influenced spouses’ opportunities to maintain relationships and showed that the availability of places that offered privacy were integral to maintaining the spousal relationship (Førsund & Ytrehus, 2018). In some cases, there was a preference for access to common areas in the nursing home as opposed to private rooms. This preference was partly because the common areas provided proximity to the staff, which was appreciated by the spouses, especially as the residents’ dementia progressed (Førsund & Ytrehus, 2018). Furthermore, earlier research has underscored the saliency of the arrangement of common areas in nursing homes for enabling person-centred care (Edvardsson et al., 2010; Fleming et al., 2016), as well as a sense of home and belonging (Johansson et al., 2022). Still, there is a scarcity of research exploring the perspectives of family members of residents regarding the physical environment of nursing homes. Little is known about family members’ perspectives on common areas in relation to the view of a nursing home as a pleasant place. For the purposes of this study, we use the term, a pleasant place, to reflect our ambition of remaining open to various ideas and aspects that family members perceive as relevant when reasoning about the common areas, as both places they visit themselves and places where their relatives live their everyday lives.

Thus, this study aimed to explore how family members of nursing home residents reason about the physical environment in common areas in nursing homes, in relation to what constitutes a pleasant place.

### Study context – nursing homes in Sweden

In Sweden, the municipalities have overall responsibility for eldercare (Swedish Institute, 2021) and according to the Social Services Act, the municipalities are assigned to promote dignity and wellbeing for older persons (Sveriges riksdag, 2001). Eldercare may be provided by both municipal and private organisations; however, it is financed by the municipalities through taxation. To be granted a place at a nursing home in Sweden, the older person’s needs are assessed, and residents are typically living with dementia and/or physical disabilities that require around-the-clock care. Approximately 75% of the nursing home residents in Sweden are living with dementia or cognitive impairment (Sköldunger et al., 2019). Nursing homes in Sweden are usually comprised of a larger building with several smaller-scale units, each unit will accommodate eight to ten persons who live in separate apartments of approximately 30–35 m^2^ (Nord, 2013). The individual apartment is rented by the resident and, except from a bed and bedside table, it is furnished with his or her own furniture and interior design. In addition to the apartment, the residents have access to common areas that are shared with other residents (Socialstyrelsen, 2017). Most of the staff are nurse aides with a two-years diploma education. Registered nurses often have the overarching responsible for daily care and there are also other multidisciplinary professionals connected to the nursing home, including occupational therapists and physiotherapists, among others.

## Methods

The study was designed as a focus group study with family members and friends of older persons who were living in nursing homes. Participating family members and friends were recruited through three nursing homes in an urban area in Sweden. We (the first and last authors) contacted the management at three nursing homes and were invited to two next-of-kin meetings that were held regularly in the nursing homes (NH I and NH III), and to one seasonal celebration that family and friends were participating in (NH II). We presented information about the study at these meetings, to recruit participants to focus group discussions. Information sheets about the study were posted on the nursing homes’ message boards, in lifts, hallways and in public areas close to the nursing homes. The information sheets provided a brief overview of the study, what participation would involve, and invited family and friends of residents to contact the first author by e-mail or telephone if they were interested in participating in the study. Thirteen family members of residents in these three nursing homes participated in the focus group discussions. Additionally, one family member to a resident in another nursing home (NH IV) who had heard about the study contacted the first author, resulting in a total of 14 participants. We invited family members and friends with the ambition of including participants who had a range of different relationships with residents at the nursing homes. However, all 14 participants were family members, including 11 children, two partners and one sibling. Hereinafter, we use the term “family members” when referring to the participants. We did not ask specifically about how long the family members, relatives had lived at the nursing home. However, the focus group discussions revealed that the family members, relatives had lived in the nursing home from approximately two months to four years. The family members also had experiences of visiting different nursing homes in connection to their relative's move to the current place, and/or when visiting other relatives.

The focus group sessions were conducted in nursing homes I and II. The original intention was to have two, consecutive focus group sessions with the same family members. For reasons of convenience, and to achieve rich data, we maintained a degree of openness regarding inclusion in the focus group sessions. Thus, a few of the family members in the first session did participate in a second session, and a few family members only participated in the second session. See Table 1 for an overview of the focus group sessions.

**Table 1.** Characteristics of each focus group session with the family members of nursing home residents.

Focus group sessions	NH where relatives of the participating family members live	Participating family members
1a	NH I	n = 5
2a	NH I	n = 4 (n = 3 who had also participated in 1a)
1b	NH II, III	n = 7
2b	NH II, IV	n = 4 (n = 3 who had also participated in 1b)
		Total: n = 14

Note. NH: nursing home

### Ethical considerations

We shared information about integrity, voluntariness and anonymity at the information meetings and in the written invitation to the focus groups. Additionally, those issues were raised at the beginning of each session. We informed the family members that they could choose to not answer questions or withdraw their participation at any time without repercussions. We collected verbal, informed consent from the family members, and this included consent to use audio recordings to document the focus group discussions. We emphasised that the researchers did not have the mandate to influence the design at the local nursing home. The study was granted ethical approval from the Regional Ethics Review Board in Stockholm (Dnr. 2015/512-31/5).

### Data generation and analysis

Basic principles for planning and conducting focus group sessions were used in the data generation (Morgan & Kreuger, 1997). This methodology was used to encourage interactions among the family members and to generate unique, shared experiences which provided rich data related to the study aim (Kitzinger & Barbour, 1999). The first and last authors were both present during all focus group sessions. The focus group discussions were moderated by the last author and lasted for approximately two hours each. The discussions were audio recorded and the moderator guided discussions towards topics that were aimed to elicit the family members’ reasonings about what constitutes a sense of a pleasant place in a nursing home. We used several tools, such as photographs and other objects, to avoid consolidating normative or stereotypical beliefs associated with nursing home environments in relation to pleasant places. The moderator emphasised that the aim was not to evaluate the environment at the specific nursing home and encouraged the participants to contribute and share their experiences from various places to the discussion. Consequently, the family members referred to the nursing home where their relatives lived, to other nursing homes that they had visited, and to other public places in the discussions.

In focus group sessions 1a and 1b, the moderator initially asked how the family members experienced the room where the focus group took place, in relation to what they perceived as a pleasant place. Furthermore, the moderator asked questions that addressed where and how the family members used to socialise with their relatives when they visited the nursing home. These questions were intended to elicit discussions that focused on situations in the physical environment, in relationship to a pleasant place, in a concrete way. Secondly, we provided a variety of photographs that were chosen to prompt vivid discussions beyond expectations related to pleasant (or unpleasant) nursing home environments. The photographs illustrated examples of situations and activities that capture different types of interactions, interior details or different locations related to the everyday life in common areas in nursing homes. The family members were encouraged to choose a few photographs and to present and discuss the place on the chosen photograph in relation to a pleasant place. To identify topics for further discussion in the following sessions (2a and 2b), we summarised the first focus group sessions (1a and 1b) based on the audio files and notes taken to capture our immediate reflections shortly after the focus group sessions. Additionally, towards the end of the first focus group sessions (1a and 1b) the family members were asked to bring their own photographs or objects that reflected what they considered could represent a pleasant place in general to the second sessions (2a and 2b). This prompted further discussions related to nursing homes as pleasant places. We started the second session (1b and 2b) by capturing the participants´ reflections from the first session, which also worked as an inviting gesture to the participants that did not participate in the first session. This generated spontaneous vivid discussions about experiences, preferences and suggestions related to the nursing home as a pleasant place. Further on in the discussion in session 1b, we presented a variety of objects chosen to prompt discussions (Schaeffer & Palmgren, 2017). The objects included for example a piece of sheepskin, stones, pliers, and headache medicines. The family members were encouraged to choose a few objects and discuss them in relation to positive values that had been identified in the preliminary analysis of the earlier session, including belonging and a sense of a pleasant place. Our intention was to use objects in the same way in session 2b, however, the discussions evolved as rich and lively and we judged that introducing objects would interrupt the discussions.

Data from all focus group sessions were analysed using qualitative content analysis (Graneheim et al., 2017; Graneheim & Lundman, 2004; Krippendorff, 2004). As an initial step, the first author transcribed the audio-recorded data from the focus group sessions 1a and 2a verbatim. The transcribed text was read several times, in parallel with repeated listening to the audio files to keep the analysis close to the original data. Inspired by Graneheim and Lundman (2004), the first and the last author identified meaning units related to the study aim. The meaning units were labelled with a code which captured the included meaning and these were discussed with the second author. The codes were continuously compared with each other to find substantive similarities and differences related to the purpose of the study, and codes with similar meanings were merged into preliminary themes. As a second step, we expanded the analysis to include focus group sessions 1b and 2b. The first and last authors listened to the audio-recorded data from focus group sessions 1b and 2b and took extensive notes, guided by the preliminary themes from focus group sessions 1a and 2a. The themes were refined through an iterative process that included repeated listening to all audio files and analytical discussions among the first, second and last authors. To remain close to the original data throughout the different levels of abstraction in the analysis process, we alternated the analysis between the whole and sections of the transcribed interviews (Graneheim et al., 2017).

## Findings

The following four themes were identified in relation to the research aim: (i) keeping a door ajar; (ii) why does it have to be so ugly?; (iii) a place to care for?; and (iv) allegiance to the place. During the focus group discussions, there was an evocative use of language to convey “ugly”, “boring”, or “cosy” spaces and artefacts, as well as a sense of care, or lack of care, for the nursing home physical environment. Family members discussed how concrete aspects of the building and interior were interconnected to the more abstract values related to experiencing pleasant and meaningful moments in everyday life.

### Keeping a door ajar

Keeping a door ajar refers to the family members’ descriptions of recognising and valuing potential opportunities within the nursing homes and with the outside world. The family members saw the presence of keeping a door ajar as away to value seemingly insignificant and yet potentially meaningful moments of everyday life. This is illustrated in the following quote from a family member, who described a situation where the nursing home staff helped to keep a door ajar to the surrounding community. The staff member had supported the resident to perform an errand which the resident perceived as meaningful:

Instead of just running across the street to the [grocery store] herself [the staff] to buy a stamp, she brought my mother there, so that my mother could use her own purse and pay. Oh, she was so happy the whole day.

(Focus Group [FG]-1b)

The focus group discussions emphasised the importance of nursing homes to provide opportunities for residents to have access to the surrounding community. The family members presented different examples of how this could be realised through keeping a door ajar. This did not necessarily mean that the residents literally went outside the nursing home building, as family members also discussed the importance of being able to “see people come and go”, which was even discussed as a human right. The participants discussed how views from the windows or balconies could serve to keep a door ajar to participate in the changing seasons and nature, as shown by the following quote:

When it is summer, he sits on the balcony from morning till evening. He knows each bird’s nest. He knows everything, he sees the buds, and when they start budding, and he knows when autumn is about to start.

(FG-1a)

Keeping a door ajar also refers to characteristics of the indoor spatiality that provided opportunities for a variety of actions, activities, events, and atmospheres. For example, access to large common areas was discussed as desirable to keep a door ajar to social events, such as traditional celebrations or seasonal parties, as it allowed for larger social group gatherings and for individuals who used wheelchairs. Events that took place in larger common areas also provided opportunities for relatives and friends to share pleasant moments with residents and staff at the nursing homes. Alternatively, keeping a door ajar to smaller spaces could foster cosiness and an intimate atmosphere which was also discussed as valuable.

A door could be kept ajar to experiences of participation in small-scale and ongoing aspects of everyday life at the nursing homes. For example, family members of residents living at the same nursing home unit described how the placement of a sofa in a corridor, adjacent to various spaces, including the entrance, private rooms, corridors, and the kitchen, kept a door ajar for residents to gather in an informal place that offered a sense of community and belonging to the unit. In the quote below, the family members reflected upon how the placement of the sofa created a sense of keeping a door ajar to pleasurable everyday moments without requiring any special arrangements from either the staff or the residents:

P1: [….] when my father moved in, there was a sofa right inside the door. There were always a few people sitting there, also my father. This was because you could see into the kitchen [….] see who was coming in the corridor, who came from the lift.

P2: Get sort of an overview

P1: Yes, exactly. It was a bit like sitting outside a café or like in the Mediterranean. You sit there by the café and nothing particular is happening, but you just stay there, like that.

(FG-2a)

The theme, keeping a door ajar, highlights how the family members reasoned about the importance of spatial conditions to provide potential opportunities for pleasurable everyday moments or special events. Importantly, keeping a door ajar illustrates that it was not necessary that such opportunities were taken and transformed into actions. The valuable aspect was the potentiality of the opportunity which contributed to a pleasant everyday life by generating a sense of an ongoing life, both within the nursing homes and with the outside world.

### Why does it have to be so ugly?

When reasoning about what characterises physical environments that may be perceived as pleasant or unpleasant, the family members, with frustration, raised the question “why does it have to be so ugly?”. When the family members discussed ugliness in general terms, the moderator asked if they could clarify their reasoning about what they considered to be “ugly” in nursing homes. In response to this question, the family members provided examples related to the overall physical environment, as well as to specific spaces and artefacts. The examples included, donated or left-over furniture from residents that had passed away and curtains that were too short. The family members associated “ugly” nursing home environments with an atmosphere which they described as institutional or impersonal. In response to different photographs related to nursing home environments provided by the moderator, the family members agreed that a consistent and well-composed environment was crucial for a nursing home environment to be perceived as pleasant. However, they sometimes had different opinions and preferences regarding style and design.

The family members elaborated on explanations of the ugliness and concluded that it stemmed from a prioritisation of logics and values related to institutional efficiency, rather than creating a pleasant and welcoming place. Institutional efficiency was discussed in relation to materials and artefacts which could be easily maintained and that were associated with cost efficiency. For example, the awkward length of the curtains was presented as exemplifying ugliness, and this was attributed to the priority of making it easy to clean the floor beneath the curtains without the curtains getting wet. The topic of floor materials and rugs was brought up repeatedly in the focus group discussions. The family members understood the floor material to have been chosen based on the logics and values of cost efficiency as well as effective maintenance, and they suggested alternatives to what they described as the “awful linoleum floors”. When discussing alternatives, the family members mentioned aspects of effective maintenance, for example: “the material has to withstand repeated cleaning after people have urinated on it”. They also discussed cost efficiency in terms of the cost and sustainability of the material, and one family member suggested that a real wooden floor could withstand hundreds of years of sweeping. Discussions partly aligned with the logics and values of cost efficiency and effective maintenance, but the family members suggested that those logics were not necessarily in conflict with logics of creating a pleasant and welcoming place if materials and products were carefully selected.

Overall, the focus group discussions highlighted the family members’ perceptions of how institutional logics guided the design of the nursing home environments, and how this resulted in spaces and artefacts that were regarded as “ugly”. Ugliness evoked connotations of an institutional or impersonal environment as a whole and this was considered contradictory to the sense of a pleasant and welcoming place.

### A place to care for?

The theme, a place to care for?, captures how the focus group discussions emphasised the importance of arranging and using common areas in nursing homes with care in order for them to be perceived as pleasant places. The theme, aplace to care for?, was often discussed in relation to examples of a lack of care, such as neglected and fragmented common areas that signalled a lack of dignity for the residents. The family members repeatedly used the term “boring”, which captured an imprecise sense of the atmosphere and implied that the place was not taken care of. The family members described details in the physical environment that suggested that the place was not cared for, including “boring” colours, plants or objects, that were displayed or left without a sense of purpose or context, as illustrated by the following quote:

P1: … it is also this, that there is an old doll laying on a cupboard, a corner cupboard, [….] I have never seen anybody using it, and a big beach ball. Maybe they have used it sometime, but they do not need to be there all the time, in a living room.

Moderator: The sense that things are just left and forgotten?

P1: It gives a feeling of a lack of dignity. It is an unpleasant feeling in a way. Does it have to be like this?

P2: left and forgotten, just as you said

(FG-1a)

A recurring topic in the different focus groups was the role of televisions (TVs) in the orchestration of activities in the common areas. Through the focus group discussions, the family members agreed that when a TV was used with care it could contribute to the creation of a pleasant and welcoming atmosphere. Using a TV with care was described as staff carefully selecting when to turn a TV on, and which programmes and/or channels to choose. Lack of care when using a TV was exemplified by situations where residents were sitting in their wheelchairs for hours in front of a TV which was constantly on, and situations when a centrally placed TV could dominate a whole room and thus, prevented contact between those who wanted to socialise with other residents and/or with visiting friends and relatives.

The theme, a place to care for?, illustrates that family members perceived that an environment that was cared for, in terms of being carefully arranged and used with sensitivity, was an important aspect of a pleasant place.

### Allegiance to the place

Through the analysis, we discovered how the family members’ attitudes towards the nursing homes could be characterised by loyalty and a sense of belonging to the places where their relatives lived. We identified this attitude as an allegiance to the place. According to the family members, the physical environment of the nursing homes did have shortcomings; however, these shortcomings were somewhat diminished by their positive experiences related to valuable aspects of the nursing homes. These positive experiences served as the basis for their allegiance to the nursing homes, as exemplified by the following quote:

Even though I don’t think this is the most cosy place in the world, this is what we chose. It is safe. This is where we are.

(FG-1a)

Allegiance to the place can be understood as being connected to the family members’ commitment to their own decisions and choices that resulted in their relatives moving into a particular nursing home. The “right choice” was confirmed by the family members’ experiences of the nursing home as providing conditions for a sense of security and belonging for their relatives, but also for themselves, as illustrated with the following quote:

We looked at five, or six nursing homes before we made our choice. Some of them were, uh, so boring so you got the chills. There was just a table and some chairs in some corner by some window. It was cold, yes completely cold. But here, you could feel it as soon as you come in, this is a place that we can choose.

(FG-1a)

Allegiance to the place was often communicated in connection with the family members’ discussions about how they experienced a sense of relief and peace of mind when their relatives moved to the nursing home, after a period of anxiety and stress over their relatives’ gradually deteriorating health and difficulties managing everyday life in their ordinary homes. This is shown in the following quote:

For me and my sister, it was like heaven when our mother could move here.

Before she lived in her own apartment with home help. It didn’t work out at all. When she could come here, the sense of happiness was mainly for us, the relatives. The staff here, they took care of the food, medicines and all of that, so there was a relief from the worries and burdens for us.

(FG-1b)

Allegiance to the place also related to the family members’ experiences of a sense of belonging to the nursing home community, that included themselves as well as their relatives, other residents, and staff. The focus group discussions pointed to how the arrangement of spontaneous and/or routinised, larger- and smaller-scale activities and events contributed to a sense of belonging to the nursing home community. One family member talked about how a member of the nursing home staff spontaneously arranged a karaoke session, as an example of a situation in the common areas which created a sense of belonging and community:

I came one evening, it was just a few days ago, and I heard someone who was singing [….] in the TV room. She was singing, you know Elvis Presley and the Evergreens, and I felt […] tears came in my eyes… We were maybe not everyone [at the unit], but everyone who was out of bed. We were six, seven [people …]. It was magic, completely magic.

(FG-2b)

Other situations that included singing, dancing, and listening to music together, were described as life-affirming and as contributing to shared experiences among the family members and the wider nursing home community. The family members discussed how such events affected everyone present, regardless of their relationships, health conditions or cultural backgrounds.

The focus group discussions included many examples of how the family members contributed to the environment in the common area, and as such to the shared nursing home community. These included examples of family members taking their dogs to the unit, others provided weekly newspapers to the common areas, or invited all residents for shared coffee and cake when they celebrated birthdays and other occasions. The family members described that those contributions to the environment were appreciated by many of the residents. In this sense, the family members contributed to the creation of a pleasant place, as members of the nursing home community (Table 2).

**Table 2.** Summary of the findings.

Themes	Brief explanation	Examples
Keeping a door ajar	Family members reasoned about the importance of spatial conditions to provide potential opportunities for pleasurable everyday moments or special events	The family members discussed how views from the windows or balconies could serve to keep a door ajar to participate in the changing seasons and nature
Why does it have to be so ugly?	When family members referred to spaces and artefacts as ugly, they explained and questioned how ugliness resulted from institutional logics that differed from the logics of creating a pleasant place	The family members understood the floor material to have been chosen based on the logics and values of cost efficiency as well as effective maintenance, and they suggested alternatives to what they described as the “awful linoleum floors”
A place to care for?	Family members perceived that an environment that was cared for, in terms of being carefully arranged and used with sensitivity, was an important aspect of a pleasant place	The family members described details in the physical environment that suggested that the place was not cared for, including “boring” colours, plants or objects, that were displayed or left without a sense of purpose or context
Allegiance to the place	Despite their recognition of shortcomings in the nursing homes’ physical environments, the family members expressed loyalty and a sense of belonging to the places where their relatives lived	The focus group discussions pointed to how the arrangement of spontaneous and/or routinised, larger- and smaller-scale activities and events contributed to a sense of belonging to the nursing home community that included themselves as well as their relatives, other residents, and staff

Despite the family members’ recognitions of shortcomings in the nursing homes’ physical environments, their allegiance to the place both contributed to, and resulted from, a sense of the nursing homes as pleasant and shared places.

## Discussion

The findings presented four themes: (i) keeping a door ajar; (ii) why does it have to be so ugly?; (iii) a place to care for?; and (iv) allegiance to the place, which illuminate how family members of nursing home residents reasoned about a pleasant everyday life in relation to common areas in the nursing homes. The findings contribute knowledge about: (a) family members’ perceptions of what characterises a pleasant nursing home; (b) family members’ reasoning on how and why common areas in nursing homes are designed the way they are; and (c) family members’ relationships to the nursing home where their relatives live. We discuss the findings in relation to these three areas in the following section.

### What characterises a pleasant nursing home environment?

All four themes provided knowledge about the family members’ reasoning and ideas about what characterises a pleasant nursing home environment related to the common areas. This topic has rarely been explored from the perspective of family members in previous research. Keeping a door ajar referred to a sense of potentiality that provided possibilities for spontaneous actions and participation in everyday life that is ongoing in the nursing homes’ common areas. Various researchers have argued that everyday matters, such as spontaneity and variety in everyday life, as well as having influence on, for example, when, how, and with whom to do everyday activities, are often regarded as trivial in nursing home policies and practices (Harnett, 2014; Mondaca et al., 2020). Policies and practices instead focus on organised activities, such as group activities and traditional celebrations or seasonal parties (Harnett, 2014; Mondaca et al., 2020). The findings from this present study highlight the importance of common areas that offer opportunities for a variety of actions, activities, events, and atmospheres, which are potentially meaningful moments of everyday life for family members, residents, and staff. Previous research has shown how spatial dynamics that support such variety facilitate a sense of home and belonging in nursing homes (Calkins, 2018; Johansson et al., 2022). In addition, previous research (Calkins 2018; Falk et al., 2013; Fleming et al., 2016; Johansson et al., 2022) has emphasised that environments that support opportunities for variety in everyday life may be particularly important in nursing home contexts, since nursing home residents spend most of their time within the nursing home. Førsund and colleagues have highlighted the saliency of an environment that enables visiting spouses to adapt their visits to the residents’ changing health and progressive dementia, to support the maintenance of spousal relationships (Førsund & Ytrehus, 2018; Førsund et.al., 2016).

Our findings show that the potentiality did not necessarily have to be enacted but had an intrinsic value by providing a sense of connectedness to the ongoing life within, as well as outside, the nursing homes. These findings support Bollig et al. (2016) who described how watching people through a window could promote dimensions of wellbeing for nursing home residents. Similarly, Johansson et al. (2022) found that opportunities for participating in activities from a distance may be valuable for creating a sense of home and belonging among nursing home residents. Still, the findings also illustrate the importance of prompts that supported the residents to enact the potentialities. Such prompts could include a physical environment that invites participation in different activities and places (Johansson et al., 2022), but also initiatives taken by the staff in response to indications of what a resident wanted to do. In this study, this was illustrated in the example of a staff member who accompanied one resident to a grocery store to buy a stamp.

The theme, why does it have to be so ugly, addressed aspects of the physical environment that conflicted with a pleasant place, which also provided insights about what characterises the design of a pleasant place. For the family members, an “ugly” nursing home environment meant a physical environment that communicated an impersonal and institutional design. Earlier research suggests that the manifestation of the institutional power of nursing homes is reflected through the physical environment in nursing homes (Harnett, 2014). This aligns with the general argument that institutional aspects should be hidden to prevent an institutional atmosphere (Calkins, 2018; Klaassens & Meijering, 2015; Shield et al., 2014). However, as concluded by Calkins (2009), the term “non-institutional” is frequently used to denote quality in the design of nursing home settings but it is rarely defined. The findings from this study add some details and nuances to what non-institutional design might imply. Moreover, the theme, a place to care for?, showed how environments and objects that were handled with care were perceived to contribute to a pleasant place, which will be further discussed in the following section.

### Reasoning about how and why common areas in nursing homes are designed the way they are

To describe the common areas in the nursing homes, the family members used aesthetic value-laden terms, such as “cosy”, “ugly” or “boring”. Those terms can be understood as metaphors of sensory impressions of an atmosphere or “a place spirit” (Martin, 2002; Pink, 2004). Following Pink (2004), the aesthetic, value-laden terms can be understood to include moral judgements about how and why the situation is shaped in the way it is. Our findings suggest that the family members may answer the question, “why does it have to be so ugly?” by replying that “the nursing home management made the wrong priorities”, in other words, prioritising institutional and economic efficiency over the logics of creating a pleasant place at the nursing home. Similarly, shortcomings in the physical environment highlighted in the theme, a place to care for?, were understood by the family members as indicative of a neglected environment. In line with theories about how care can be understood in relation to materiality (Buse et al., 2018; Maller, 2015), the findings show how the care of objects in a nursing home environment is imbued with several meanings that cover a broad spectrum of how the objects can be understood and used. The value the family members ascribed to a nursing home environment that is handled with care and sensitivity can be understood as signifying whether the residents were treated in a dignified way, with care and sensitivity (Buse et al., 2018; Cleeve, 2020; Maller, 2015).

### Family members’ relationships to the nursing home where their relative lives

The theme, allegiance to the place, explicitly addressed the family members’ relationships to the nursing homes. This topic was also partly addressed in the theme, why does it have to be so ugly?, as it showed how the family members engaged in providing suggestions to “reduce ugliness” in the physical environment of nursing homes. The family members’ engagement and loyalty included the family members’ awareness of the logics that guided the design of the common areas in the nursing homes, and their efforts to understand and take those logics into account in their suggestions.

Allegiance to the place was connected to the nursing home as a whole, which included staff, residents, management, the building and also the atmosphere which was created by those aspects and actors altogether. This finding aligns with previous research that has shown how a shared culture is manifested in actions and attitudes, as well as the physical environment. Defining such shared cultures as “a beating heart”, Johansson et al. (2022) showed how this could contribute to a sense of home and belonging connected to a nursing home. Similarly, Davies and Nolan (2006) showed how family members may contribute to the nursing home community by “making it better” not only for their own relative but also for other residents and staff. Our findings highlight the importance of belonging from the perspective of family members, which has not been discussed in relation to common areas in nursing homes in earlier research. Allegiance to the place included loyalty with a nursing home and gratitude to staff which supports earlier research (Bollig et al., 2016). However, the loyalty and gratitude did not preclude family members from recognising shortcomings in the everyday practices, or organisation of the nursing homes. Rather, allegiance to the place seemed to mitigate and take precedence over the family members’ criticism of the nursing homes’ shortcomings in the common areas in relation to the physical environment.

## Methodological considerations

This study is potentially limited by the relatively small number of participants (n = 14) and the limited geographic scope of the participating nursing homes which were all located in a single urban region. The credibility of our findings is strengthened by the breadth of experiences that the family members had from different nursing homes. Thus, the family members could refer to different types of atmospheres, situations, and designs when they reasoned about pleasant nursing home environments. The participants were mainly adult children to the residents as opposed to spouses, which might have influenced the perspectives of the discussions. We recognise, however, that the participating family members may have been especially engaged in the nursing homes and that the findings are not necessarily representative of all family members. Moreover, the focus group format and use of photographs and objects, prompted vivid discussions about nursing homes in relation to pleasant places.

## Conclusion

Overall, the present study highlights family members’ emotional, as well as practical engagement in the nursing home community where their respective relatives live, with a particular focus on the physical environment in the common areas. The findings highlight the importance of spatial conditions to provide potential opportunities for keeping a door ajar to pleasurable everyday moments. Furthermore, the theme, a place to care for, emphasised the physical environment as an integrated aspect of care, and the value of caring for the physical environment, in terms of being carefully arranged and used with sensitivity. This study has taken on the challenge of grasping the everyday aspects of a pleasant place from the perspective of family members of nursing home residents. As such, this study contributes valuable knowledge for design, and redesign, of common areas in nursing homes, which includes the physical environment as an integral aspect of care and moves beyond the ideas of non-institutional environments.
